# Induction of Sperm DNA Fragmentation by Cryopreservation and In Vitro Incubation: Comparison of TUNEL, SCSA, SCD Test and COMET Assay

**DOI:** 10.3390/ijms26188978

**Published:** 2025-09-15

**Authors:** Costanza Calamai, Michele Tanturli, Donata Conti, Giorgio Leter, Linda Vignozzi, Lisa Giovannelli, Monica Muratori

**Affiliations:** 1 Department of Experimental and Clinical Biomedical Sciences “Mario Serio”, University of Florence, 50139 Florence, Italy; costanza.calamai@unifi.it (C.C.); michele.tanturli@unifi.it (M.T.); linda.vignozzi@unifi.it (L.V.); 2 Research Unit of Histology & Embryology, Department of Experimental & Clinical Medicine, University of Florence, 50139 Florence, Italy; donata.conti@unifi.it; 3 Laboratory of Health and Environment, Division of Health Protection Technologies, ENEA Casaccia Research Center, 00123 Rome, Italy; gleter110@gmail.com; 4 Andrology, Women’s Endocrinology and Gender Incongruence Unit, AOU Careggi, 50134 Florence, Italy; 5 Department Neurofarba, Section Pharmacology and Toxicology, University of Florence, 50121 Florence, Italy; lisa.giovannelli@unifi.it

**Keywords:** sperm DNA fragmentation, TUNEL, SCSA, SCD test, COMET assay, cryopreservation, in vitro incubation

## Abstract

TUNEL, SCSA, SCD test and COMET assay are the current tests for detection of sperm DNA fragmentation (sDF) in clinical practice. These four tests are very different from each other for many aspects, possibly including the type of revealed damage. To verify how the same type of damage was revealed, we simultaneously detected sDF by the four tests before and after induction of sperm DNA breakage by cryopreservation and in vitro incubation. We found that all tests revealed the increase in sDF in both experimental conditions. However, when we pairwise compared the fold increases in induced sDF, we found poor (i.e., values below 0.5) Lin’s concordance correlation coefficients (CCCs) both during cryopreservation and in vitro incubation. The only exception was for SCD test/COMET assay where the CCCs were about 0.5 (cryopreservation: 0.456 (95% CI −0.071–0.784) and incubation: 0.523 (95% CI −0.018–0.827)). Bland–Altman plot analysis showed that TUNEL reveals the highest amounts of sDF during cryopreservation, whereas LiveTUNEL indicated that such damage is undergone by the viable sperm fraction. This is the first study comparing the four tests in detection of sperm DNA damage during cryopreservation and incubation.

## 1. Introduction

Male infertility affects 7% of male population, a figure that appears to be worsening in light of two main meta-analyses published in recent years [1,2]. These studies demonstrate, in recent decades, a dramatic and still ongoing decline in sperm count, considered one of the key proxies for male fertility [3]. The causes of such decline are attributed to the deep environmental changes occurred in the same time range, including the increase in exposure to pollutants and to poor lifestyles [1]. This scenario is worsened by the fact that treatments for male infertility are quite limited as many causes are untreatable, or idiopathic (conventional semen parameters are abnormal but the cause is not known) or unexplained (conventional semen parameters are normal, and female factor can be excluded but the subject remains infertile).

A fraction of cases of idiopathic and/or unexplained male infertility might be due to high levels of sperm DNA fragmentation (sDF), consisting of single- and double-stranded DNA breaks in sperm nuclei. This type of sperm damage has been gaining the attention progressively in recent decades due to its clinical correlates. Indeed, the amounts of sDF are more able to predict natural conception [4,5] than conventional semen parameters, increase in couples with recurrent pregnancy loss [6,7] and, according to some authors [8] but not confirmed by others [9], also negatively impact the outcomes of the Assisted Reproductive Technologies (ARTs). In agreement with these findings, the last version of the WHO manual for semen analysis [10] suggested detection of sDF as an adjunct to routine semen analysis in the male infertility work-up.

Currently, four main tests are available for sDF detection in clinical practice: TUNEL (Terminal deoxynucleotidyl transferase dUTP Nick-End Labeling), SCSA (Sperm Chromatin Structure Assay), SCD (Sperm Chromatin Dispersion) test and COMET assay. TUNEL detects the 3′OH ends released by single and double breaks to DNA. SCSA detects the susceptibility of sperm chromatin to denature after a slight acidification. SCD test detects sDF as the inability of DNA fragmented sperm cells to form a halo around the nuclear core and COMET assay (alkaline version) detects the single- and double-stranded DNA fragments that migrate toward the anode during an electrophoretic run. Among these tests, the gold standard one for detection of sDF has not yet been established. On the other hand, these tests are very different from each other for many aspects, including the underlying principles, the specificity and the sensitivity of the yielded measures [11]. It is possible that the type of revealed damage is also different among the tests. TUNEL and COMET assay would reveal the real DNA breakage, whereas SCD test and SCSA would rather detect abnormalities in sperm chromatin.

This study aimed at comparing the four tests after inducing the same type of damage. To this aim, we detected sDF by the four tests in the same sperm samples before and after induction of sperm DNA damage by cryopreservation or in vitro incubation. In addition, using LiveTUNEL [12], we studied the development of sDF in viable and non-viable spermatozoa during cryopreservation.

## 2. Results

### 2.1. Induction of sDF by Cryopreservation

**Figure 1:**
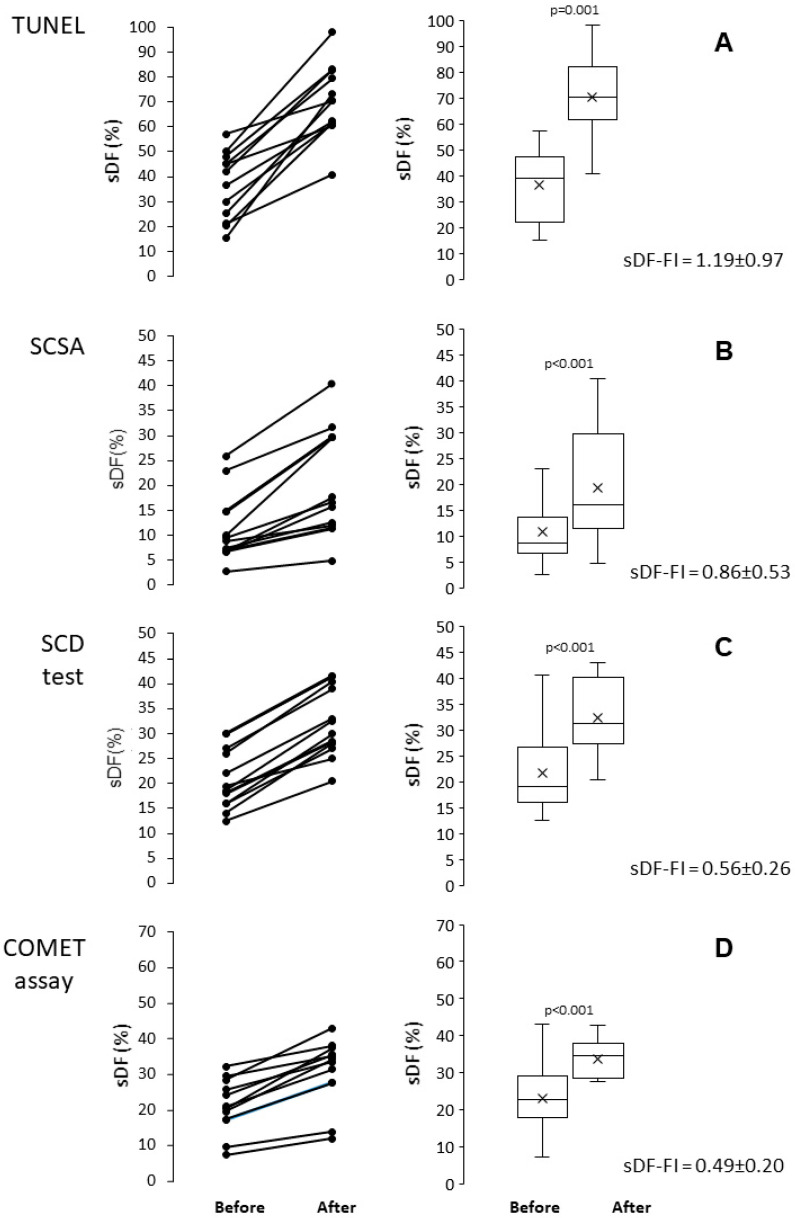
Induction of sperm DNA fragmentation during cryopreservation of human sperm, as detected by TUNEL (**A**), SCSA (**B**), SCD test (**C**) and COMET assay (**D**). Left panels: individual values of sDF before and after cryopreservation; right panels: box graphs reporting mean, median [IQR] and minimum and maximum values of sDF before and after in vitro cryopreservation. *t*-test for paired data.

In this experimental set, sDF was induced by a cryopreservation procedure consisting of Vapour Fast Freezing (VFF), using Test Yolk Buffer (TYB) as freezing medium and 500 µL straws as carriers. In 12 semen samples, sDF was detected before and after freezing/thawing with TUNEL, SCSA, SCD test and COMET assay. Figure 1 reports the individual (first column) and the median and mean values of sDF (second column) before and after cryopreservation. As shown, all tests resulted able to detect the increase in sDF induced by freezing/thawing. In Figure 1, the mean fold increase [(value after − value before)/value before] of sDF (sDF-FI) for each test is also reported.

### 2.2. Induction of sDF by In Vitro Incubation

**Figure 2:**
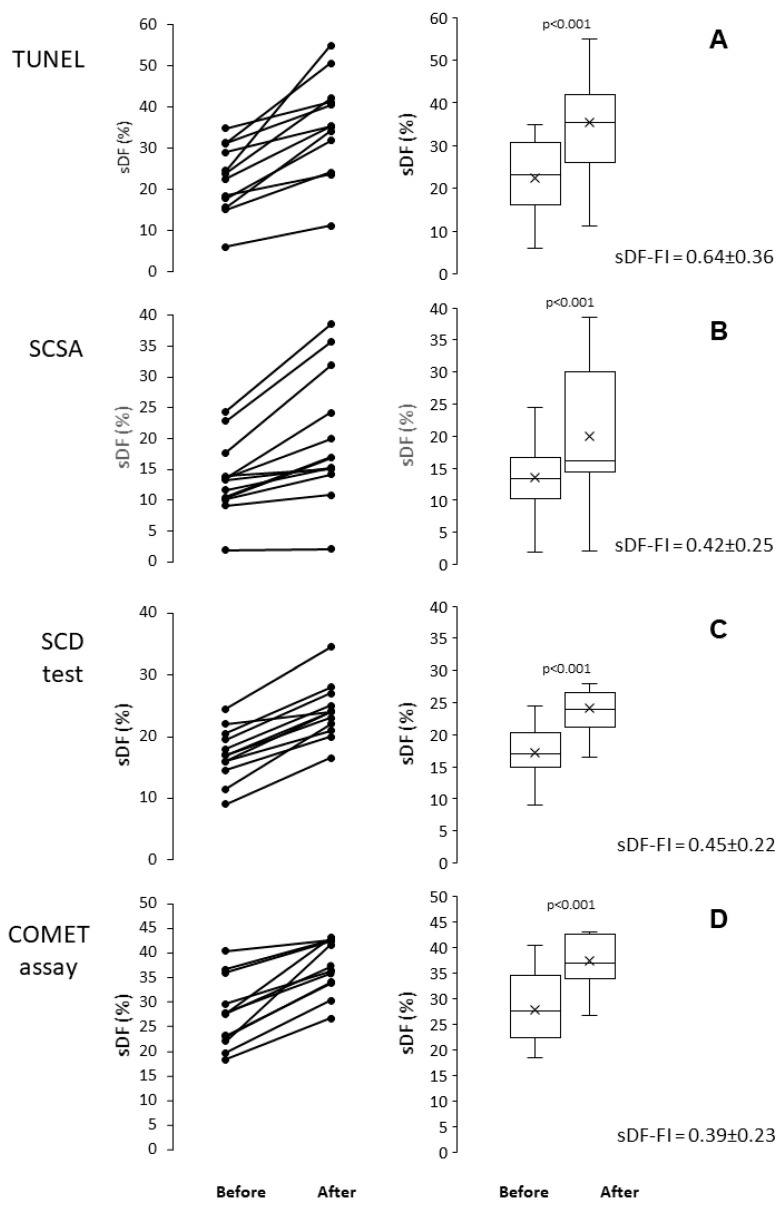
Induction of sperm DNA fragmentation during in vitro incubation of human sperm, as detected by TUNEL (**A**), SCSA (**B**), SCD test (**C**) and COMET assay (**D**). Left panels: individual values of sDF before and after in vitro incubation; right panels: box graphs reporting mean, median [IQR] and minimum and maximum values of sDF before and after in vitro incubation. *t*-test for paired data.

In this experimental set, sDF was induced by in vitro incubating sperm for 24 h, at 37 °C in a 5% CO_2_ atmosphere. In 12 sperm samples prepared by density gradient centrifugation (DGC), sDF was detected before and after incubation with TUNEL, SCSA, SCD test and COMET assay. Figure 2 reports the individual (first column) and the median and mean values of sDF (second column) before and after incubation. As shown, all tests resulted able to detect the increase in sDF induced by incubation. In Figure 2, the mean sDF-FI for each test is also reported.

### 2.3. Comparison Between Cryopreservation and In Vitro Incubation

**Figure 3:**
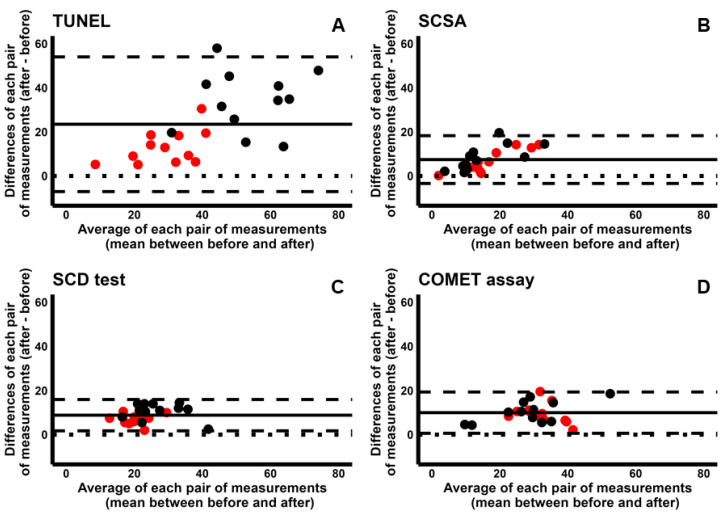
Comparison between sperm DNA fragmentation induced during cryopreservation and incubation as detected by each of the four tests: (**A**), TUNEL; (**B**), SCSA; (**C**), SCD test; (**D**), COMET assay. In the Bland–Altman plots, the difference between sDF values after and before incubation (red points) or cryopreservation (black points) are plotted against the averages of the two values. Mean difference, solid line; mean difference ± 2SD, dashed lines; differences = 0, dotted line.

For each test, we compared the increase in sDF obtained during cryopreservation and incubation. To this aim, we used Bland–Altman plots where the increases in sDF (differences between the value after and that before induction) were plotted against the averages of the two values (before and after induction) (Figure 3). Since all four tests detected the increase in sDF during both cryopreservation and incubation, all dots lie above the perfect agreement line (y = 0). However, TUNEL detects a much higher increase during cryopreservation than incubation (Figure 3A, black dots vs. red ones). This result was not present with the other tests, where the increases in sDF were similar between incubation and cryopreservation (Figure 3B: SCSA; Figure 3C: SCD test; and Figure 3D: COMET assay).

### 2.4. Pairwise Comparison Among the Four Tests

**Figure 4:**
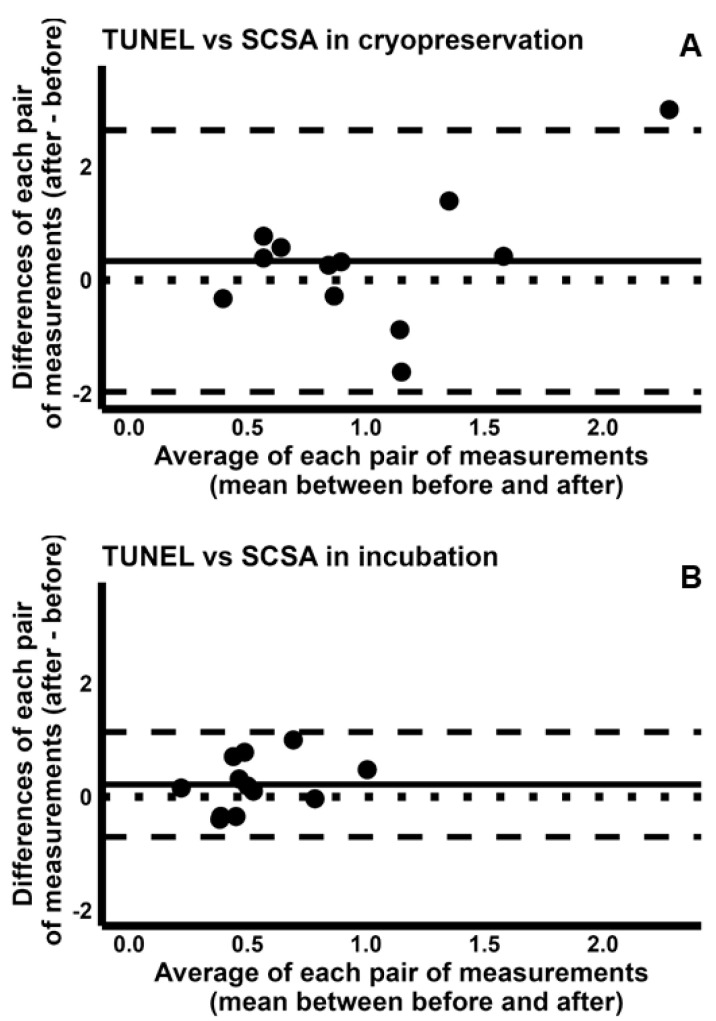
Bland–Altman plots comparing the fold increases of sperm DNA fragmentation obtained by TUNEL with those by SCSA, during cryopreservation (**A**) and incubation (**B**). The differences between the sDF-FIs obtained by TUNEL and those by SCSA were plotted against the mean of the two values. Mean difference, solid line; mean difference ± 2SD, dashed lines; differences = 0, dotted line.

To explore how differently the four tests detected the induction of sDF, we used the sDF-FIs during incubation and cryopreservation, yielded by each test. Regarding TUNEL and SCSA, we found a poor CCC both for cryopreservation [−0.080 (95% CI −0.506–0.377)] and incubation [−0.082 (95% CI −0.498–0.364)] and reported the respective concordance plots in Figures S1A and S1B, respectively. To further compare the two tests, we used the Bland–Altman plots (Figure 4), showing that the sDF-FIs detected by TUNEL are overall higher than those detected by SCSA during both cryopreservation (Figure 4A) and incubation (Figure 4B). Indeed, most dots are above the line of perfect agreement. However, the average of measures by two tests has a larger range in cryopreservation than incubation (range 0.40–2.28 vs. 0.22–1.00, respectively, Figure 4). In addition, in cryopreservation but not in incubation, for higher sDF-FI values, the difference between the two tests increases (Figure 4A). Overall, the Bland–Altman plot analysis indicates that TUNEL detects more damage than SCSA during cryopreservation, but not during incubation.

**Figure 5:**
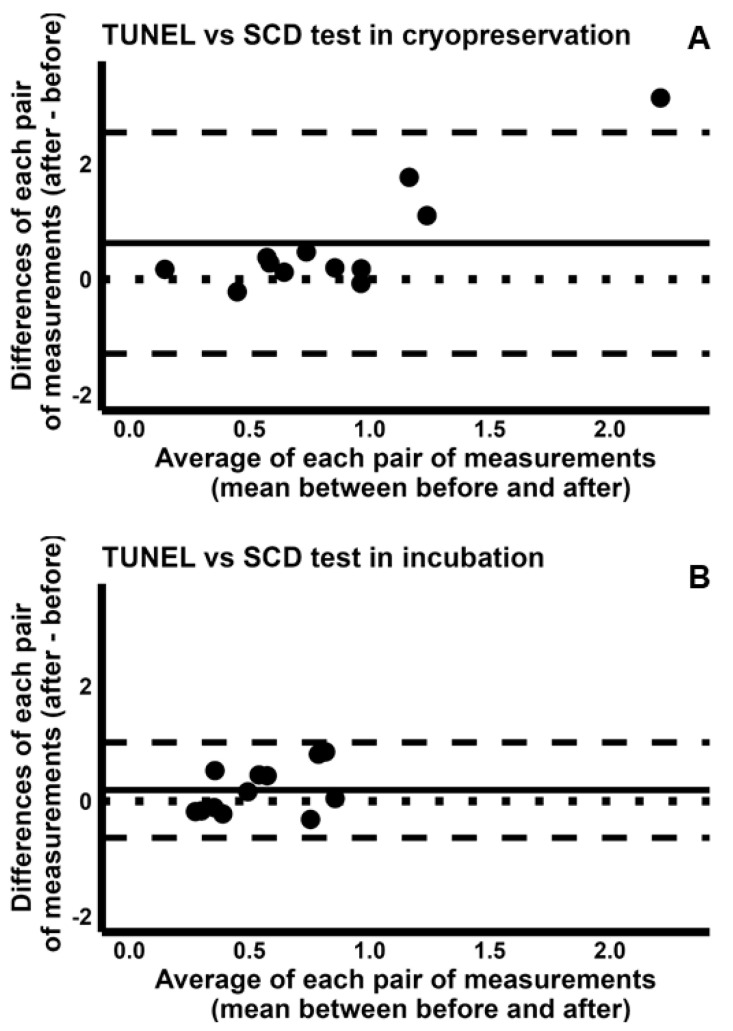
Bland–Altman plots comparing the fold increases of sperm DNA fragmentation obtained by TUNEL with those by SCD test during cryopreservation (**A**) and incubation (**B**). The differences between the sDF-FIs obtained by TUNEL and those by SCD test were plotted against the mean of the two values. Mean difference, solid line; mean difference ± 2SD, dashed lines; differences = 0, dotted line.

**Figure 6:**
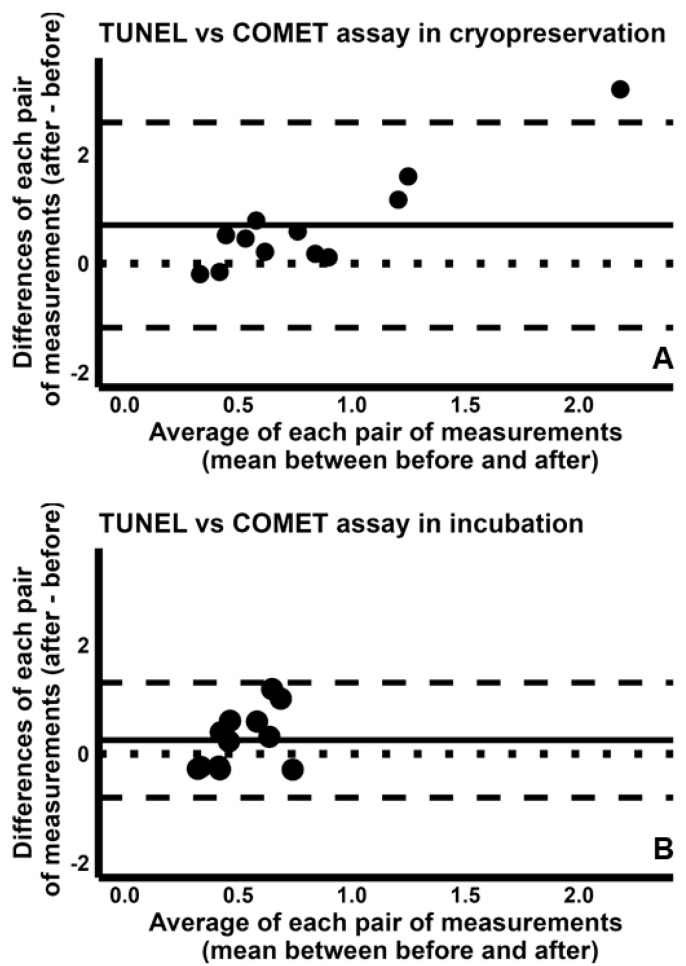
Bland–Altman plots comparing the fold increases of sperm DNA fragmentation obtained by TUNEL with those by COMET assay, during cryopreservation (**A**) and incubation (**B**). The differences between the sDF-FIs obtained by TUNEL and those by COMET assay were plotted against the mean of the two values. Mean difference, solid line; mean difference ± 2SD, dashed lines; differences = 0, dotted line.

Similar results were found when the sDF-FIs of TUNEL were compared to those of SCD test and of COMET assay. Indeed, we found poor values for CCC during both cryopreservation [TUNEL/SCD test: 0.057 (95% CI −0.157–0.267); TUNEL/COMET assay: 0.057(95% CI −0.102–0.212)] and incubation [TUNEL/SCD test: 0.028 (95% CI −0.401–0.448); TUNEL/COMET assay: −0.399 (95% CI −0.724–0.069)]. These results are reported in Figure S1 (TUNEL/SCD test, cryopreservation: Figure S1C; incubation: Figure S1D. TUNEL/COMET assay, cryopreservation: Figure S1E; and incubation: Figure S1F). The analysis by Bland–Altman plots revealed that the sDF-FIs detected by TUNEL were higher than those detected by SCD test or COMET assay during cryopreservation (TUNEL/SDC test: Figure 5A; TUNEL/COMET assay: Figure 6A), but similar during incubation (TUNEL/SDC test: Figure 5B; TUNEL/COMET assay: Figure 6B).

**Figure 7:**
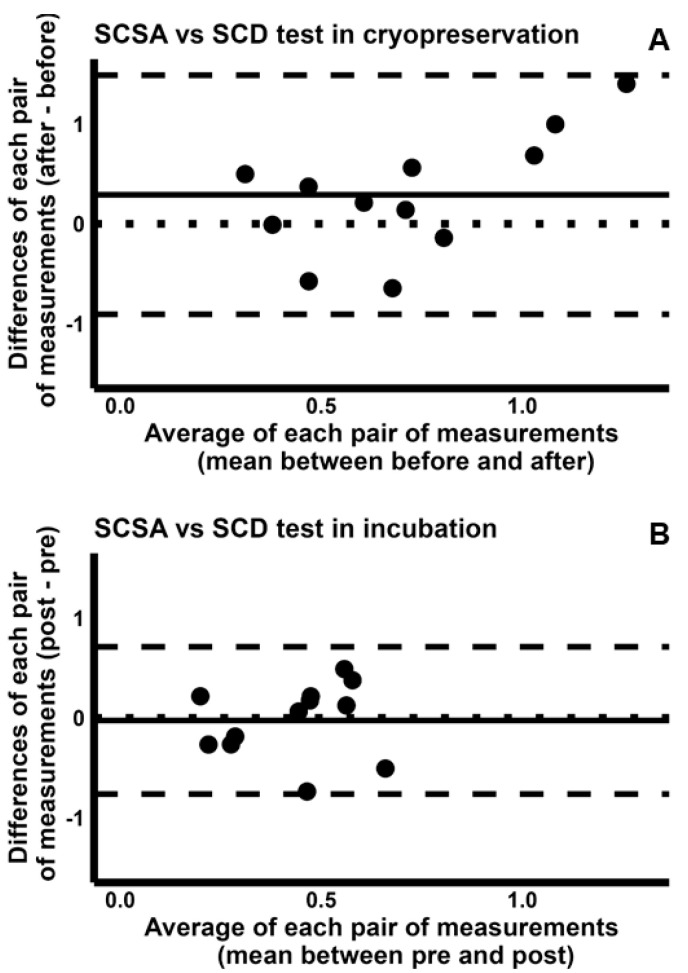
Bland–Altman plots comparing the fold increases of sperm DNA fragmentation obtained by SCSA with those by SCD test, during cryopreservation (**A**) and incubation (**B**). The differences between the sDF-FIs obtained by SCSA and those by SCD test were plotted against the mean of the two values. Mean difference, solid line; mean difference ± 2SD, dashed lines; differences = 0, dotted line.

**Figure 8:**
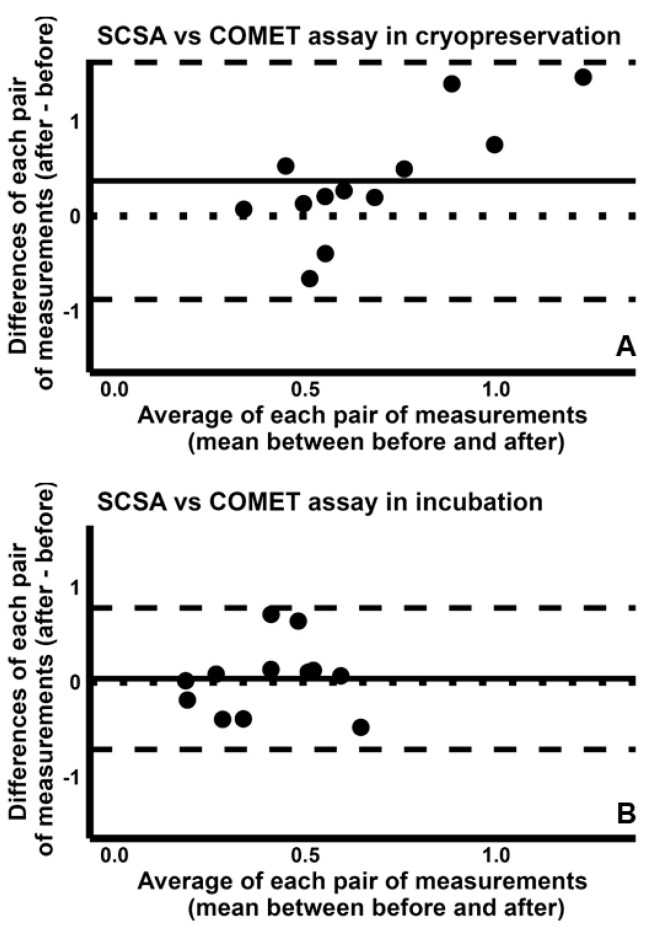
Bland–Altman plots comparing the fold increases of sperm DNA fragmentation obtained by SCSA with those by COMET assay, during cryopreservation (**A**) and incubation (**B**). The differences between the sDF-FIs obtained by SCSA and those by COMET assay test were plotted against the mean of the two values. Mean difference, solid line; mean difference ± 2SD, dashed lines; differences = 0, dotted line.

When we compared the ability of SCSA to detect induced sDF to that of SCD test and of COMET assay, we found again poor CCCs [SCSA/SCD test, cryopreservation: −0.015 (95% CI −0.379–0.352); incubation: −0.188 (95% CI −0.664–0.397). SCSA/COMET assay, cryopreservation: −0.136 (95% CI −0.399–0.147); incubation: −0.198 (95% CI −0.669–0.387)]. Concordance plots are reported in Figure S2 (SCSA/SCD test, cryopreservation: Figure S2A; incubation: Figure S2B. SCSA/COMET assay, cryopreservation: Figure S2C; incubation: Figure S2D). The Bland–Altman plots indicated that the sDF-FIs detected by SCSA during cryopreservation are higher than those detected by both SCD test (Figure 7A) and COMET assay (Figure 8A), although such difference was not so sharp as in the comparison between TUNEL and these two tests. Conversely, detection of sDF induced during incubation was similar between SCSA and SCD test (Figure 7B) or COMET assay (Figure 8B).

**Figure 9:**
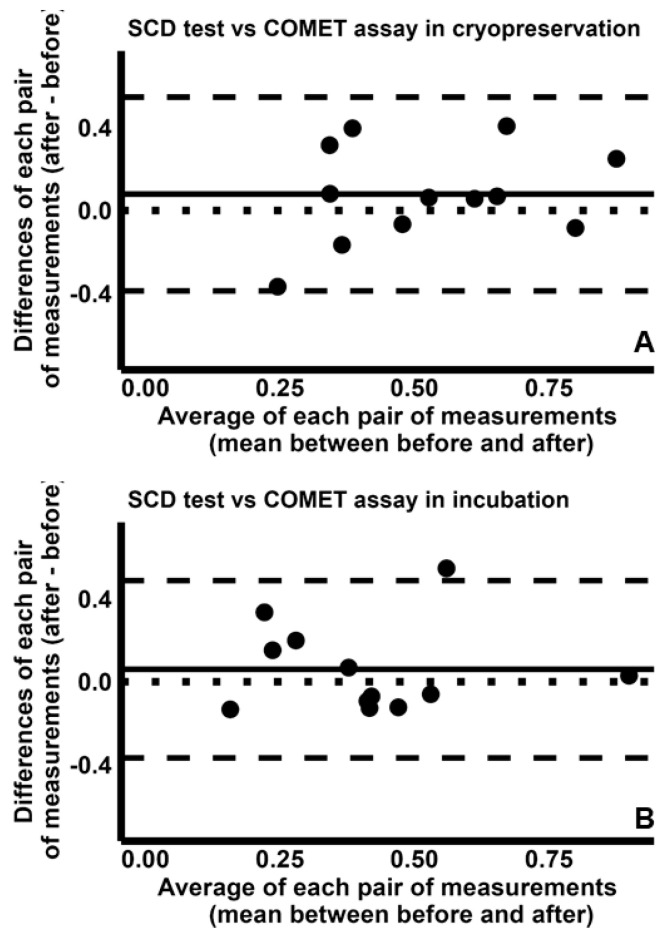
Bland–Altman plots comparing the fold increases of sperm DNA fragmentation obtained by SCD test with those by COMET assay, during cryopreservation (**A**) and incubation (**B**). The differences between the sDF-FIs obtained by SCD test and those by COMET assay test were plotted against the mean of the two values. Mean difference, solid line; mean difference ± 2SD, dashed lines; differences = 0, dotted line.

Finally, comparison between SCD test and COMET assay indicated fair CCC values [cryopreservation: 0.456 (95% CI −0.071–0.784); incubation: 0.523 (95% CI −0.018–0.827)], with concordance plots reported in Figures S3A (cryopreservation) and S3B (incubation). Further, the Bland–Altman plot analysis suggests that the two tests are similar in detecting the increases in sDF during both cryopreservation (Figure 9A) and incubation (Figure 9B).

### 2.5. LiveTUNEL Before and After Cryopreservation

**Figure 10:**
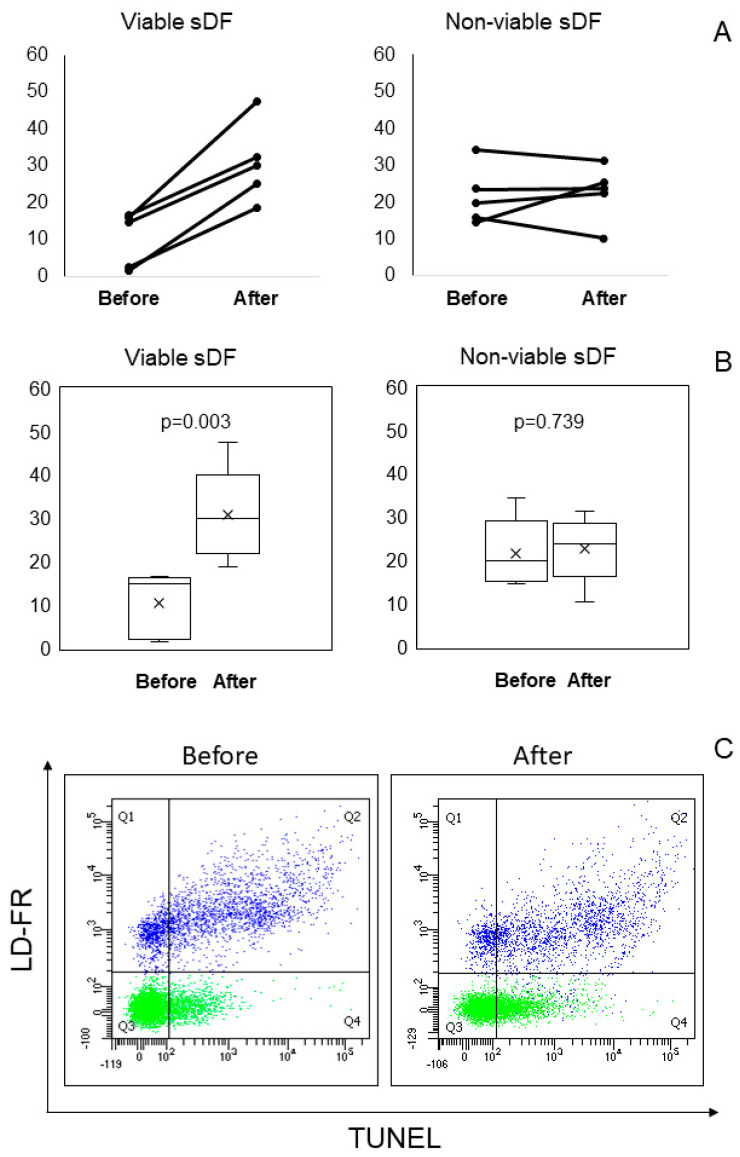
Induction of sperm DNA fragmentation in viable and non-viable sperm during cryopreservation, as detected by LiveTUNEL. (**A**). Individual values of viable and non-viable sDF before and after cryopreservation. (**B**). Box graphs reporting mean, median [IQR] and minimum and maximum values of viable and non-viable sDF before and after cryopreservation. (**C**). Typical LIVE/DEAD Fixable Far Red Dead Cell Stain (LD-FR)/TUNEL dot plots reporting DNA fragmentation in viable (green) and non-viable (blue) sperm. Left panel: before cryopreservation; right panel: after cryopreservation.

As shown, during cryopreservation TUNEL appears to detect an additional damage with respect to both incubation and other tests. To verify whether such damage was due to viable or non-viable sperm fraction, we used LiveTUNEL [12] in five semen samples. Figure 10 reports the results observed before and after cryopreservation (Figure 10A, individual values; Figure 10B, median and mean values of sDF), and the LiveTUNEL dot plots of a typical experiment (Figure 10C). As shown, in all samples, cryopreservation induced an increase in DNA damage in viable spermatozoa, whereas in the non- viable ones the amounts of sDF remained unchanged, except for one sample.

## 3. Discussion

This study compared four main tests to reveal sDF in terms of detection of sperm DNA breakage induced during cryopreservation and in vitro incubation. Our results indicated a poor pairwise concordance in detecting the induced damage, except for SCD test/COMET assay showing however fair CCCs. In addition, the analyses with Bland–Altman plots showed that the damage induced by cryopreservation is higher than that by incubation, but only when sDF is detected by TUNEL. Further, TUNEL detects higher amounts of induced sDF than each other test, but only during cryopreservation, as during incubation the four tests detect similar amounts of damage.

Previous studies mainly compared the tests by detecting sDF in native semen samples [13,14,15,16,17,18]. Native sDF can be derived from three main mechanisms that are different from a molecular point of view and that differently concur to the amount of sperm DNA damage in semen: testicular abortive apoptosis, defect during sperm chromatin maturation and oxidative attack [11]. Hence, this model is not the best way to compare tests that might preferentially detect sperm DNA breaks, depending on their molecular origin. In this context, we reasoned that simultaneous detection of an in vitro-induced damage (by cryopreservation or in vitro incubation) by the four tests would be a more effective strategy to address whether the four tests reveal differently a same type of damage and thus whether these tests also differ for the type of revealed damage. Indeed, during in vitro sperm manipulation, the only mechanism supposed to induce sperm DNA breaks is oxidative attack [19,20] and thus the induced damage should be of the same type.

To measure the induced damage, we used the fold increase vs. the basal (before cryopreservation/in vitro incubation) value. Indeed, this parameter is not affected by difference in test sensitivity, making it a more effective measure of each test’s ability to detect damage in the two experimental sets. To pairwise compare the sDF-FIs of the four tests, we used the Lin’s CCC, a coefficient that accounts for not only the strength of the relationship between the two measures, but also the closeness to perfect agreement. As mentioned, the type of the induced damage by cryopreservation (or incubation) was supposed to be the same. Nevertheless, the CCC values for the pairwise comparisons were rather poor, except for SCD test/COMET results, where the CCC value was about 0.5. Hence, apart from SCD test/COMET, the four tests appeared to detect the same induced damage rather differently. A similar result was reported for SCSA and SCD test in dog sperm incubated for 24 h [21]. Indeed, using the Intraclass Correlation Coefficient, this study concluded that the two tests detect “different aspects of DNA fragmentation” [21].

The pairwise Bland–Altman plot analyses of sDF-FIs obtained by four tests indicated that, during cryopreservation, TUNEL reveals higher amounts of induced sDF than other tests. At a lesser extent, in frozen-thawed samples, SCSA also detected higher increases in sDF than SCD test and COMET assay, whereas similar results among the tests were observed during incubation. Conversely, SCD test and COMET assay yielded similar results during both cryopreservation and incubation.

To our best knowledge, few studies used more than one test to reveal the damage induced during cryopreservation [22,23,24,25,26,27,28] or incubation [21], reporting differences among tests. In cats, SCD test revealed tenfold amounts of sDF with respect to SCSA [22], and non-correlating values between these two tests were also reported in cryopreserved bull sperm [23]. The poor ability of SCSA to detect damage induced by freezing/thawing was also shown in a study in humans, where this test was compared to TUNEL [24]. Further, according to several studies in humans [25] or rams [26,27], after cryopreservation, TUNEL coupled with fluorescence microscopy yields lower amounts of sDF than COMET assay, although one of the studies conducted in ram reported opposite results [28]. Overall, these results show little consistency with our study. However, we have to consider that all these studies compared tests using the sDF amounts obtained after induction of damage, thus also depending on the sDF amounts before induction and by test sensitivity. The latter, in turn, is greatly affected by the used procedure for each test. Indeed, except for SCSA, these tests exist with many variants yielding different results in the same samples [29,30]. In this scenario, a direct comparison between previous studies and our results cannot be performed.

As mentioned, TUNEL detects the highest amounts of damage induced by cryopreservation with respect to other tests. In addition, when we compared, within each test, the sDF values after induction of sperm DNA versus those before, we found that with TUNEL, but not with the other tests, the increases in DNA damage were higher during cryopreservation than in vitro incubation. These results prompted us to speculate that cryopreservation induces an additional type of damage that is detected above all by TUNEL. It has been reported that TUNEL is a test preferentially detecting DNA breaks in peri/post mortem sperm [31,32,33]. Since cryopreservation highly decreases sperm viability [19,34,35], we reasoned that the higher amounts detected by TUNEL with respect to both incubation and the other tests could be due to non-viable cells. To test this hypothesis, we revealed sDF before and after cryopreservation with LiveTUNEL, able to detect DNA breakage in viable and non-viable sperm. However, contrary to our hypothesis, the obtained results showed that the sperm fraction that mainly undergo DNA breaks is the viable one, whereas in the non-viable one sDF remains nearly unchanged. Hence, if during cryopreservation TUNEL detects an additional damage with respect to other tests and incubation, such damage is due to the viable sperm fraction.

Results of this study might also be of importance in clinical practice, particularly in the ART setting as oocyte insemination is conducted with selected/incubated or frozen/thawed spermatozoa. Although natural conception is predicted by the sDF level irrespective of the test used to reveal it [14,36], results of meta-analyses on the impact of different tests on assisted reproductive outcomes are contradictory. Some authors reported that tests such as TUNEL and COMET assay better predict pregnancy rates in couples treated by in vitro fertilisation (IVF)/Intracytoplasmic Sperm Injection (ICSI) than the other tests [8,37]. Conversely, others found no effect of sDF on this outcome, not even when subgrouping studies by tests for sDF detection [9]. On the other hand, very few studies have directly compared two or more tests in terms of assisted reproductive outcomes [15,38,39,40]. One of these studies, conducted with COMET assay and TUNEL, found no effect of sDF on fertilisation rate and embryo quality and no difference in sDF amounts was observed between ICSI cycles that resulted in pregnancy and those that did not [38]. Similarly, in bovines, neither COMET assay nor SCSA detected a difference in sperm DNA damage between animals with poor and good embryo development [39]. Conversely, Simon et al. reported lower values of sDF in pregnant couples treated by IVF/ICSI than those in non-pregnant ones, but only when TUNEL and COMET assay were used, as SCSA failed to detect such difference [15]. However, in a similar type of couples, both SCSA and SCD test did detect a negative impact of sDF on the fertilisation rate and live birth rate [40]. Overall, it is clear that further studies directly comparing the available tests for sDF detection in terms of assisted reproductive outcomes would be necessary to assess which test is the most suitable in ARTs.

In conclusion, this is the first study comparing the four tests for sDF detection that are currently used in the clinical practice. In particular, with these tests, we simultaneously detected the same type of damage, i.e. that induced by freezing/thawing processes and by in vitro incubation, and compared the obtained sDF-FIs. Our results indicated that the tests are quite different each other, as indicated by the very low to fair CCCs. We also found that the version of TUNEL used in the study revealed the highest increases in damage induced by cryopreservation. Although it is not clear which type of damage is preferentially detected by TUNEL during cryopreservation, such damage is developed within viable cells. Overall, results of this study might be important for ART setting where cryopreservation and in vitro incubation are spready applied.

## 4. Materials and Methods

### 4.1. Reagents and Media

Human Tubal Fluid (HTF), TYB and Human Serum Albumin (HSA) were purchased by Fujifilm (Rome, Italy). A Halosperm kit was obtained from Halotech DNA (Madrid, Spain). Acridine orange (AO), LD-FR and SYBR™ Gold Nucleic Acid Gel Stain (SYBR Gold) were purchased from Thermo Fisher Scientific (Waltham, MA, USA). All other reagents were obtained from Merck Life Science (Milan, Italy), unless otherwise indicated.

### 4.2. Sample Collection

Semen samples were collected by male partners of infertile couples referred to the Semen Cryopreservation and Andrology Laboratory of Careggi Hospital to execute routine semen analysis. Semen sample collection and routine semen analysis were conducted according to the WHO guidelines [10]. We excluded subjects with the following: (i) azoospermia; (ii) an insufficient sperm number for simultaneously detecting sDF with the four tests before and after cryopreservation or in vitro incubation (see below); (iii) leukocytospermia; (iv) semen viscosity; (v) bacteriospermia. Average values for age, abstinence and main semen parameters of recruited subjects for the study are reported in Table S1.

### 4.3. Cryopreservation

Native semen samples were split into 4 aliquots: (i) 8 million-cell aliquot, immediately processed using TUNEL; (ii) 100,000-cell aliquot, immediately processed by SCD test technique; (iii) 500,000-cell aliquot, stored at −80 °C and shipped on dry ice to the flow cytometry (FCM) facility of the Section of Toxicology and Biomedical Sciences (ENEA Casaccia, Rome, Italy) for SCSA analysis; (iv) 30,000-cell aliquot, stored in liquid nitrogen for later analysis using COMET assay. Preliminary results indicated that the COMET assay conducted in snap frozen samples yielded similar values of sDF as fresh samples (respectively: 22.36 ± 5.83% versus 22.77 ± 5.68%; n = 5, *p* > 0.05, *t*-test for paired data). The remaining sample was cryopreserved with a VFF procedure. Briefly, semen samples were diluted (1:1, vol:vol) by dropwise addition of TYB and kept at 37 °C for 15 min for equilibration. Then, the mixture was aspirated into 500 μL high-security sperm straws. After sealing, the straws were placed in liquid nitrogen vapour for 8 min and then plunged into liquid nitrogen (−196 °C) for storage. Samples were thawed quickly at 37 °C and then washed with 500 µL of HTF medium, centrifuged and resuspended in 1 mL of the same medium. The latter was split into four aliquots, one for each test as described above.

### 4.4. In Vitro Incubation

Sperm samples were prepared by DGC by layering 1 mL of semen upon a 40:80% SpermGrad gradient and centrifuging at 600× *g* for 15 min. After recovering the 80% pellet by resuspending in 1 mL of HTF-HSA 1%, the sample was centrifuged for 10 min at 200× *g* and finally resuspended in the same medium. Hence, sperm viability and concentration were determined. Samples that, after selection with DCG, did not show the required number of spermatozoa or 60% vitality were discarded. Each sample was split in 4 aliquots as described in the previous paragraph. The remaining sample was incubated in vitro for 24 h in HTF medium at 37 °C and 5% CO_2_ at a concentration of 10 million/mL. After incubation, the samples were again split into four aliquots, one for each test as described in the previous paragraph.

### 4.5. TUNEL

TUNEL was conducted with the flow cytometric version TUNEL/PI [41], using the in situ Cell Death Detection Kit, fluorescein. Semen samples were washed twice with HTF medium and then fixed with 4% paraformaldehyde (200 μL for 30 min at RT). Fixed samples were again washed twice with PBS/1%BSA and then permeabilized with 0.1% Triton X-100/sodium citrate (100 μL for 4 min in ice). After washing twice, samples were split into two aliquots that were incubated in 50 μL of labelling solution (supplied by the kit), containing (test sample) or not containing (negative control) the terminal deoxynucleotidyl transferase (TdT) enzyme (1 h at 37 °C in the dark). After incubation, samples were twice washed and resuspended in 400 μL PBS and sperm nuclei were stained with 10 μL of propidium iodide (PI, 30 mg/mL in PBS, 10 min at RT in the dark). After fluorescence compensation, sperm samples were acquired by flow cytometry (FACScan flow cytometer, Becton Dickinson, Franklin Lakes, NJ, USA). Green fluorescence due to DNA fragmentation was revealed with an FL-1 (515–555 nm wavelength band) whereas red fluorescence due to nuclear staining with PI was detected with an FL-2 (563–607 nm wavelength band) detector. For each sample, we recorded 10,000 events within an FSC/SSC flame-shaped region (F) containing both spermatozoa and semen apoptotic bodies [41]. Hence, sDF was determined after gating sperm (PI positive events) within the F region [41]. For data analysis, we set a marker in the TUNEL axis of the dot plot of the negative control, excluding about 1% of total events. After copying such marker in the dot plots of corresponding test sample, all events beyond it were considered as DNA fragmented sperm and expressed as percentage on total sperm population.

### 4.6. SCSA

For SCSA analysis, we followed a procedure previously reported [42]. Briefly, semen aliquots were quickly thawed in a 37 °C water bath and immediately treated with an acid–detergent solution (0.08 N HCl, 0.15 mol/L NaCl, 0.1% Triton-X 100; pH 1.2) for 30 s. Hence, samples were stained with AO (6 mg/L in a phosphate-citrate buffer, pH 6.0) and, after 3 min, analysed by a FACSCalibur flow cytometer (Becton Dickinson, Franklin Lakes, NJ, USA). Green and red fluorescence of AO were revealed with the FL-1 and FL-3 detector, respectively. We recorded a total of 10,000 events for each sample, keeping the flow rate at ∼200 cells/s. After gating sperm in the green/red fluorescence dot plot, data analyses were carried out by using SCSASoft, version 1 (SCSA Diagnostics, Brookings, SD, USA), calculating the ratio between the red and total (red plus green) fluorescence intensity (DFI, DNA Fragmentation Index) for each cell. SDF was expressed as percentages of DNA fragmented sperm and calculated from the DFI frequency histogram [43] as the fraction of sperm outside the main cell population.

### 4.7. SCD Test

We performed this test as previously reported [44] by resuspending 50,000 sperm in 1% low melting point agarose and then layering them on pre-coated agarose slides. Then, slides were covered with a coverslip and placed at 4 °C until solidification. Hence, samples were treated with an acid denaturation solution and then with a lysing solution, both provided by the Halosperm kit. After dehydration with 70% and then 100% ethanol, samples were stained with eosin and then thiazine (each for 15 min at RT), provided by the kit. Spermatozoa were examined by bright field microscopy for halos and sDF was expressed as percentage of spermatozoa without or with small halo on total spermatozoa (at least 200 cells per sample) [44].

### 4.8. COMET Assay

COMET assay was conducted with the alkaline version according to Simon and Carrell [45], with some modifications. After quick thawing at 37 °C, semen samples (30,000 sperm cells) were mixed with 1% low melting point agarose (200 µL) and two aliquots (29 µL) for each sample were layered on a GelBond film (Euroclone S.p.A, Milan, Italy). Hence, samples were covered with a coverslip and kept at 4 °C for 10 min to solidify. Then, samples were treated with a lysis solution [2.5 m NaCl, 100 mm EDTA and 10 mm Tris (pH 10), 1% Triton X-100] for 1 h at 4 °C. For chromatin decondensation, we treated samples with 10 mM dithiothreitol for 30 min at 4 °C and then we added 4 mM lithium diiodosalicylate for a further 90 min at RT. DNA alkaline unwinding was conducted in a solution containing 300 mM NaOH, 1 mM EDTA, pH 13, for 20 min at 4 °C, and electrophoresis was subsequently carried out in the same solution for 15 min at 4 °C (applied voltage: 0.8 V/cm). At the end, slides were drained, washed three times in a neutralisation buffer (0.4 M Tris, pH 7.5) for 10 min, briefly washed in water and finally dried overnight in oven at 37 °C. The next day, nuclei were stained with SYBR Gold (1:5000 diluted) and kept in a dark, moist chamber at 4 °C until microscopic analysis was performed. For image analysis, we used a fluorescence microscope equipped with a camera (Nikon Labophot-2 epifluorescence microscope) and a dedicated software (COMET IV software, Perceptive Instruments). SDF was determined as the amount of fluorescence in the comet tail (calculated as percentage of total DNA fluorescence) and expressed as a mean value in 100 randomly selected sperm nucleoids (50 for each duplicate).

### 4.9. LiveTUNEL

LiveTUNEL couples TUNEL and staining with LD-FR, allowing to detect sDF both in viable (viable sDF) and non-viable (non-viable sDF) cells [12]. This procedure was conducted, as previously reported [12], in samples before and after cryopreservation. Briefly, semen samples (8 million/mL) were washed twice with HTF medium, stained by LD-FR (diluted 1:10,000, in 500 µL of PBS) and incubated for 1 h at RT, in the dark. After further washing, the samples were fixed, permeabilized and labelled by TUNEL as described above. At the end of TUNEL, samples were stained with DAPI (1 µg/mL, 15 min in the dark at RT) until acquisition with a FACSCanto II flow cytometer (BD Biosciences, Franklin Lakes, NJ, USA), equipped with a violet laser at 405 nm, a blue laser at 488 nm and a red laser at 633 nm. To detect blue (DAPI), green (FITC), and far red (LD-FR) fluorescence, we used the 450/40, 530/30 and 660/20 BP filters, respectively.

Data acquisition and analysis were conducted with the BD FACSdiva Software (v9.0, BD Biosciences, Franklin Lakes, NJ, USA). For each sample, we acquired 8000 viable spermatozoa, i.e. LD-FR-negative events in the flame-shaped F region containing spermatozoa and semen apoptotic bodies [41].

For data analysis, within F, we gated DAPI-labelled events (i.e., spermatozoa). Hence, quadrants were set in the LD-FR/TUNEL dot plot of the negative controls (TdT omitted) and then copied in the corresponding test samples. Viable and non-viable sDF was calculated as percentages of total sperm population.

### 4.10. Statistical Analyses

Data were analysed with Statistical Package for the Social Sciences (SPSS 29) for Windows (SPSS, Inc., Chicago, IL, USA) and with R: A Language and Environment for Statistical Computing (4.5.1), using DescTools and blandr packages. Data were expressed as mean ± SD and/or median (interquartile range). To compare values of sDF obtained before to those after cryopreservation (or incubation), for each of the four tests, we used the *t*-test for paired data. A *p* value < 0.05 was considered as statistically significant. To express the increase in sDF during cryopreservation or incubation, we used the sDF-FI. Fold increases obtained by the four tests (during cryopreservation or incubation) were compared, by using the following: (i) Lin’s concordance correlation coefficient (CCC), which combines measures of both precision and accuracy to determine how far the observed data deviate from the line of perfect concordance (that is, the line at 45 degrees on a square scatter plot); (ii) the Bland–Altman plot, where the differences between values from two tests are plotted against the averages of values from the two tests. In this plot, horizontal lines represent the mean difference and the limits of agreement (the mean difference ± 1.96 times the standard deviation of the difference). Further, y = 0 is the line of perfect agreement. For each test, to compare detection of sDF during cryopreservation and incubation, we used again the Bland–Altman plot.

## Data Availability

Data are available on request from the corresponding author.

## Supplementary Materials

The following supporting information can be downloaded at: https://www.mdpi.com/article/10.3390/ijms26188978/s1.
