# Supplementary material for: Induction of Sperm DNA Fragmentation by Cryopreservation and In Vitro Incubation: Comparison of TUNEL, SCSA, SCD Test and COMET Assay
Source: Int J Mol Sci. 2025 Sep 15;26(18):8978. doi: 10.3390/ijms26188978 (PMC12469900; doi:10.3390/ijms26188978)
Supplement: Supplementary file 1 [file ijms-26-08978-s001.zip › Supplementary Table S1.pdf]

Supplementary Table S1. Age, abstinence and main semen parameters in patients recruited in the study (n=24).

**Age (y)**	31.00[22.00-39.50]
**Abstinence (d)**	4.00[3.00-5.00]
**Volume (ml)**	3.95[3.28-5.30]
**pH**	7.80[7.60-7.95]
**Concentration (10^6/ml)**	82.80[71.33-98.50]
**Number (10^6/ejaculate)**	360.50[238.76-513.74]
**Progressive Motility (%)**	61.00[52.00-74.75]
**Total Motility (%)**	66.00[58.00-77.75]
**Normal Morphology (%)**	5.00[3.25-6.75]
