# Supplementary figures and images for: Induction of Sperm DNA Fragmentation by Cryopreservation and In Vitro Incubation: Comparison of TUNEL, SCSA, SCD Test and COMET Assay
Source: Int J Mol Sci. 2025 Sep 15;26(18):8978. doi: 10.3390/ijms26188978 (PMC12469900; doi:10.3390/ijms26188978)

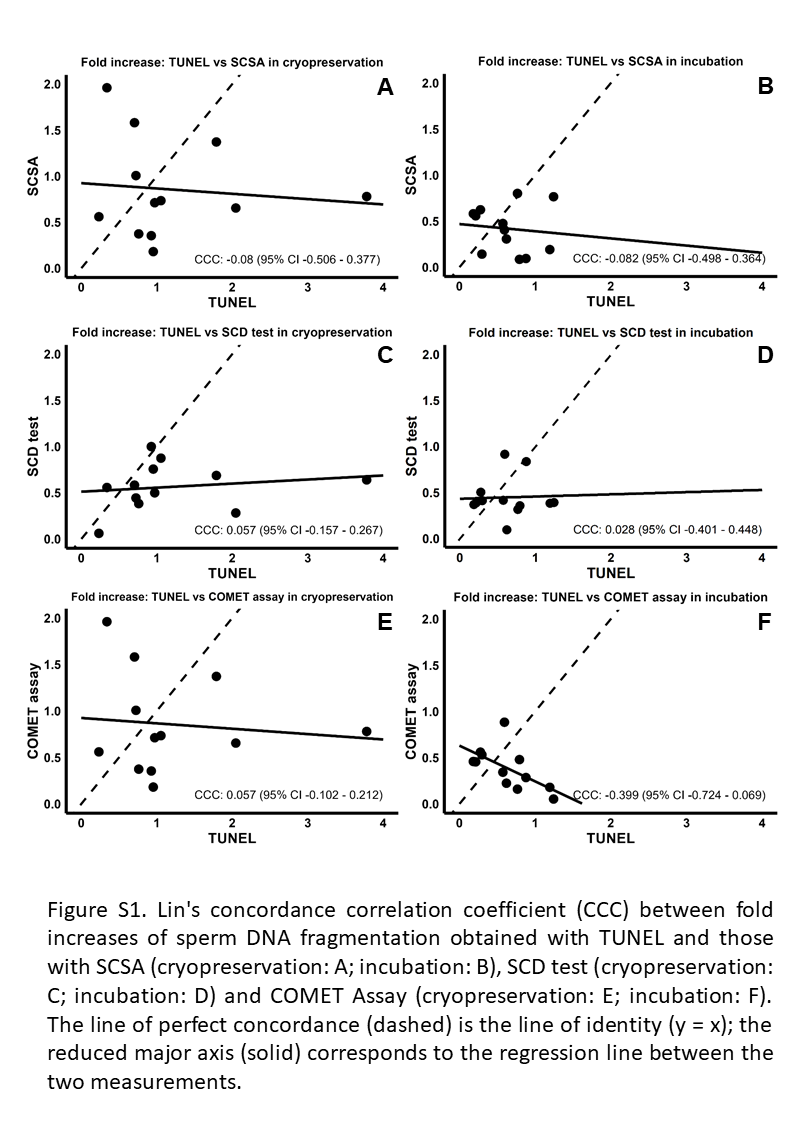

Supplement: Supplementary file 1 [file ijms-26-08978-s001.zip › Revised Figure S1.tif]

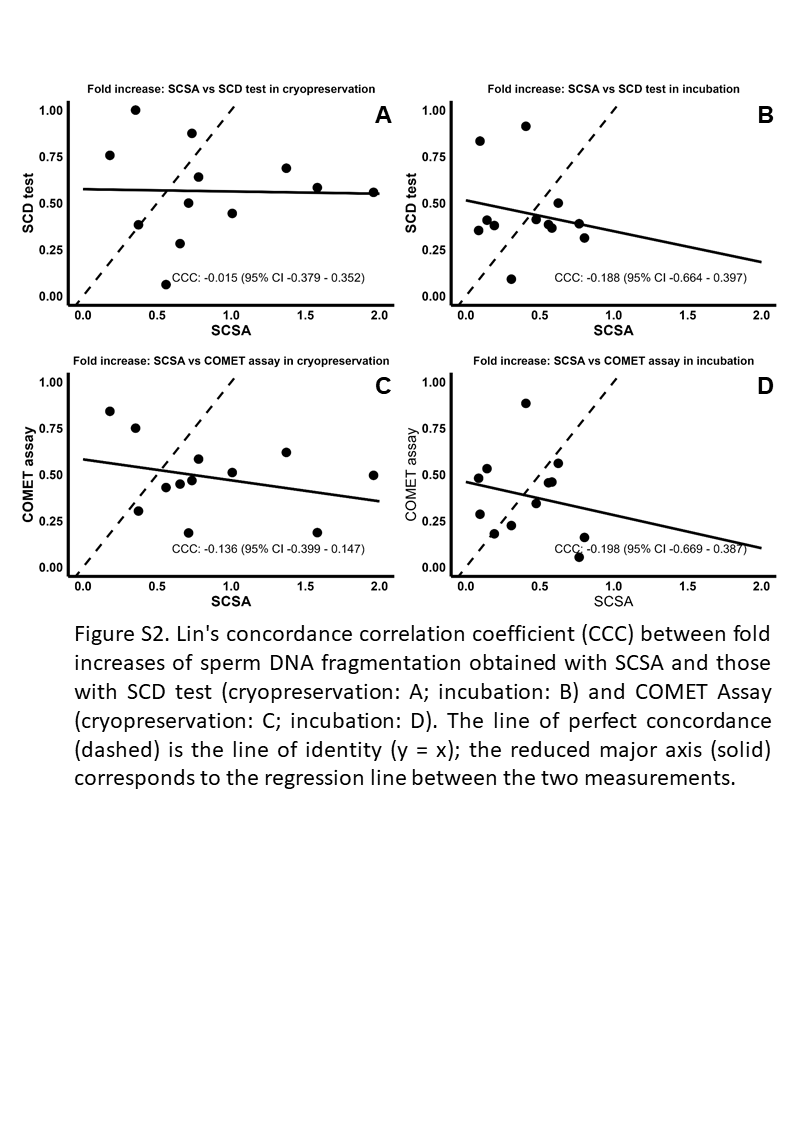

Supplement: Supplementary file 1 [file ijms-26-08978-s001.zip › Revised Figure S2.tif]

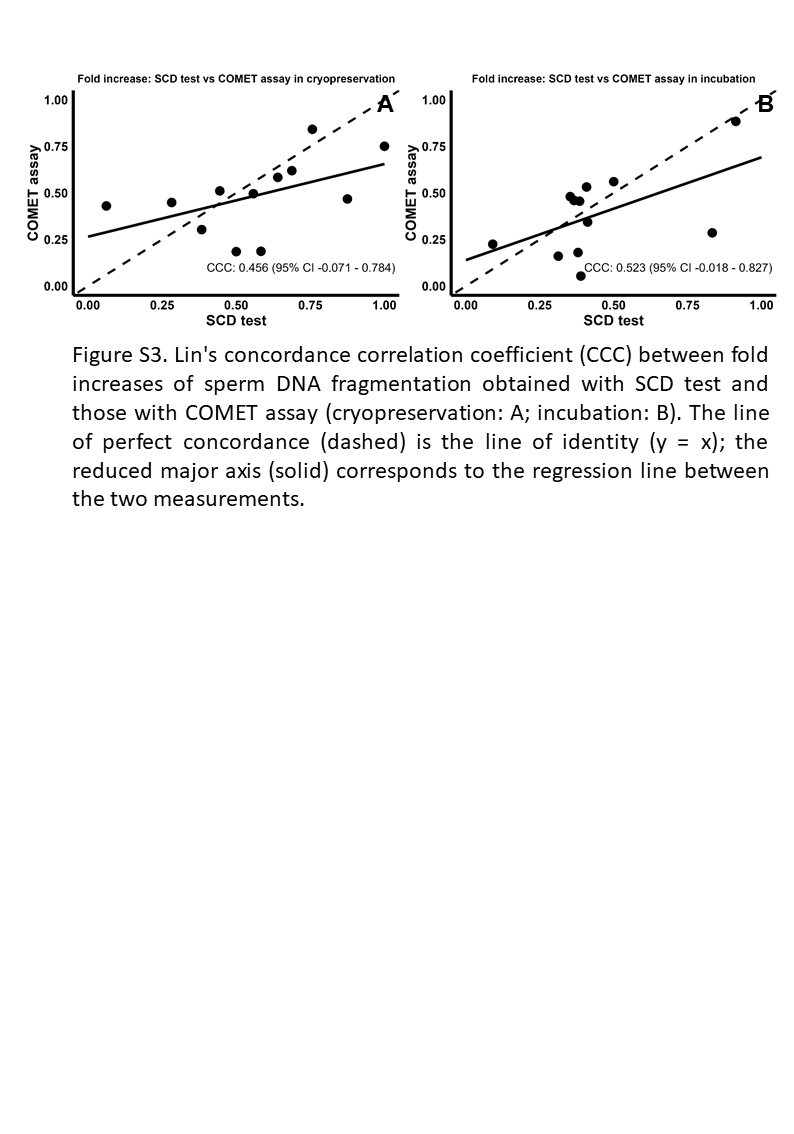

Supplement: Supplementary file 1 [file ijms-26-08978-s001.zip › Revised Figure S3.tif]
